# Effectiveness of immersive virtual reality‐based interventions for alleviating peri‐ and post‐procedural pain and anxiety in gynecologic settings: A systematic review and meta‐analysis of randomized controlled trials

**DOI:** 10.1002/ijgo.71095

**Published:** 2026-05-25

**Authors:** Cristina González‐Lara, Marina Piñar‐Lara, Esteban Obrero‐Gaitán, Héctor García‐López, Irene Cortés‐Pérez

**Affiliations:** ^1^ Asociación Engloba, Integración Laboral, Educativa y Social Jaén Spain; ^2^ ASPAYM Jaén Spain; ^3^ Department of Health Sciences University of Jaén Jaén Spain; ^4^ CAIT APROMPSI Cazorla Spain; ^5^ Department of Nursing, Physiotherapy and Medicine University of Almería Almería Spain

**Keywords:** anxiety, gynecologic procedures, immersive virtual reality, meta‐analysis, obstetrical and gynecologic, pain, virtual reality

## Abstract

**Background:**

Non‐pharmacological and complementary therapeutic, notably immersive virtual reality‐based interventions (IVR), could alleviate pain and anxiety associated with gynecologic procedures.

**Objective:**

To evaluate the effectiveness of IVR in reducing peri‐ and post‐procedural pain and anxiety in gynecologic procedures. Secondarily, to determine the safety of IVR.

**Search Strategy:**

A systematic review with meta‐analysis was conducted, after searching in PubMed/Medline, SCOPUS, Web of Science, CINAHL Complete and PEDro from database inception to September 2025 with no language or publication date restrictions set.

**Selection Criteria:**

Inclusion criteria were based on the PICOS framework: Population (patients undergoing gynecologic procedures), Intervention (IVR), Comparison (standard care or other therapeutic approaches), Outcomes (pain, anxiety and adverse events) and Study design (randomized controlled trials [RCTs]).

**Data Collection and Analysis:**

Pooled effects were calculated using Cohen's standardized mean difference (SMD) and its 95% confidence intervals (95% CI) in a random‐effects model. Potential adverse events during IVR exposure were also compiled.

**Main Results:**

A total of 25 randomized controlled trials (RCTs), comprising data from 2438 women (mean age 35.3 ± 12.6 years), were included. Intraprocedural outcomes indicated that IVR significantly decreased both pain (SMD = −0.46, 95% CI: −0.71 to −0.21, *P* < 0.001) and anxiety (SMD = −0.33, 95% CI: −0.63 to −0.03, *P* = 0.031). Post‐procedurally, IVR yielded further reductions in pain (SMD = −0.81, 95% CI: −1.12 to −0.5, *P* < 0.001) and anxiety (SMD = −0.76, 95% CI: −1.22 to −0.3, *P* = 0.001). Reported adverse events were minor and infrequent.

**Conclusion:**

This meta‐analysis suggests that IVR serves as a highly effective and safe non‐pharmacological adjunct for mitigating peri‐ and post‐procedural pain and anxiety in gynecologic settings. Translating these findings into clinical practice could substantially enhance patient care and overall experience.

****PROSPERO** Registration:**

CRD420251000835

## 1 INTRODUCTION

Pain, defined as an unpleasant sensory and emotional experience associated with actual or potential tissue damage or described in terms of such damage, is the most prevalent symptom in gynecologic clinical practice, manifesting as an unavoidable consequence of invasive gynecologic and obstetric procedures.^1^ Because effective pain management is a fundamental human right and essential for procedural optimization,^2^ addressing the physiological and psychological impacts of uncontrolled pain remains a priority for healthcare professionals.^3^ Therefore, integrating complementary treatments to reduce peri‐ and post‐procedural pain and anxiety is critical. Satisfactory pain control for women undergoing office gynecologic procedures is decisive for both patient comfort and procedure success.^4^ Consequently, it is essential to identify preventive and supportive strategies to alleviate pain and anxiety, promoting women's health.^5^ Non‐pharmacological approaches have shown promise in this regard; for example, pre‐procedural videos significantly lower anxiety and boost patient satisfaction.^6^, ^7^ Additionally, music interventions further improve perioperative outcomes.^8^, ^9^

Virtual reality (VR)‐based therapy has emerged as a complementary approach to alleviate pain^10^ and anxiety associated with gynecological procedures has emerged as a complementary approach for alleviating pain and anxiety associated with gynecologic procedures. Immersive VR (IVR) utilizes advanced software and specialized hardware (such as head‐mounted displays), motion sensors or hand‐tracking systems, to immerse users in virtual and realistic environments.^11^ IVR provides the highest levels of immersion, presence, and multisensory stimulation, being considered as the most appropriate modality of VR for pain management.^12^ The effectiveness of IVR has been shown for chronic pain conditions such as musculoskeletal pain,^13^ neuropathic pain,^14^ and fibromyalgia syndrome.^15^ During medical interventions, IVR serves as a powerful distraction tool, thereby enhancing comfort and potentially reducing reliance on opioids.^16^ Furthermore, it can be used for educational purposes with patients, due to familiarizes patients with clinical procedures and providing information, which collectively mitigates procedural anxiety and pain.^17^, ^18^ Within the field of women's health, IVR has been used to reduce pain during childbirth,^19^ decrease anxiety during fertility treatments,^20^ and assist in the management of endometriosis‐related chronic pelvic pain.^21^

Employing IVR devices as a distraction approach to mitigate discomfort during gynecologic procedures could carry substantial clinical benefits. To date, five systematic reviews with meta‐analyses (SRMAs) have evaluated the therapeutic and distractive potential of IVR devices for pain and anxiety management across diverse gynecologic and obstetrics interventions, including hysteroscopy^22^, ^23^, ^24^ and episiotomy.^25^, ^26^ However, while previous SRMAs suggest that IVR may be effective in managing pain^22^, ^24^, ^25^, ^26^ and anxiety,^22^, ^23^, ^25^, ^26^ their findings must be interpreted with caution. These reviews are constrained by the low number of included studies (ranging from 5 to 17), limited overall sample sizes (between 327 and 578 participants), and the inclusion of heterogeneous studies, mixing randomized controlled trials (RCTs) and non‐RCTs.^23^, ^25^ Consequently, the generalizability and external validity of their results can be compromised. To address these limitations and provide a meaningful contribution to the scientific literature, the present SRMA focuses exclusively on RCTs. Furthermore, this SRMA goes beyond isolated interventions by comprehensively evaluating various outpatient and surgical gynecologic procedures together. This comprehensive approach overcomes the limitations of previous literature, yielding the most robust, up‐to‐date, and generalizable evidence regarding the clinical effectiveness of IVR. Therefore, based on an exhaustive literature search, the primary objective of this study was to evaluate the effectiveness of IVR as a non‐pharmacological intervention to reduce pain and anxiety among women undergoing gynecologic procedures. A secondary objective was to examine the safety of IVR as a therapeutic tool in the context of these procedures.

## 2 METHODS

This SRMA was conducted in accordance with the Preferred Reporting Items for Systematic Reviews and Meta‐analyses (PRISMA)^27^ and the Cochrane Handbook for Systematic Reviews of Interventions.^28^ The overall methodological quality was assessed using the AMSTAR 2 checklist.^29^ This SRMA was registered in PROSPERO (CRD420251000835). Changes respect the initial register has been amended in PROSPERO.

### 2.1 Literature search and databases

Two authors (CGL and EOG) independently searched, from inception to September 2025, in PubMed Medline, Scopus, WOS, CINAHL Complete and PEDro. No language or publication date restrictions were applied. Additionally, supplementary searches were conducted by screening the reference lists of relevant published studies, congress abstracts, and Google Scholar. Eligibility criteria were defined according to the PICOS framework^30^: Population (patients undergoing gynecologic procedures); Intervention (IVR); Comparison (usual care or therapies excluding IVR); Outcomes (pain, anxiety and adverse events); and Study design (randomized controlled trials [RCTs]). The search strategy combined medical subject headings, such as “gynecologic surgical procedures,” “obstetric surgical procedures,” “virtual reality,” and “virtual reality‐based therapy” and entry‐terms using the Boolean operators “AND” and “OR” (Table S1).

### 2.2 Study selection

Two authors (CGL and EOG) independently screened the titles and abstracts of all studies retrieved from the literature search. Inter‐rater agreement was calculated using Cohen's kappa coefficient (*κ*).^31^, ^32^ Any disagreements were resolved through consultation with a third author (ICP). To be included in this SRMA, studies had to meet the PICOS criteria (previously named). Additionally, RCTs were required to report extractable quantitative data for the outcomes of interest to conduct the meta‐analyses.

### 2.3 Data extraction

Data extraction was performed independently by two authors (CGL and EOG), using a standardized form. Any discrepancies were resolved by a third author (ICP). The following data were extracted: (1) Overall characteristics (authorship, publication date, country, blinding, setting and funding); and sample demographics (number of groups, sample size per group, and age). (2) IVR characteristics (hardware and software and application protocol). (3) Comparison intervention details (type of the intervention and application). Regarding the outcome variables, we extracted the variable name, the measurement tool employed, the evaluation time‐points (during and post‐procedure), and the statistical data required for the meta‐analysis (means and standard deviations). Furthermore, the authors recorded the incidence and type of any adverse events reported by participants using the IVR.

### 2.4 Variables

The evaluated outcomes included pain intensity and anxiety. Additionally, any adverse events were documented.

### 2.5 Assessment of risk of bias and quality of evidence

These assessments were independently performed by two authors (MPL and HGL), with any disagreements resolved through consultation with a third author (EOG). First, the risk of bias for the included RCTs was evaluated using the Cochrane Risk of Bial (CRoB) tool. This tool comprises seven domains assessing selection, performance, detection, attrition, reporting, and other biases.^33^ Second, the certainty of evidence for each meta‐analyzed outcome was assessed using the Grading of Recommendations Assessment, Development and Evaluation (GRADE) statement^34^ and the Meader checklist.^35^ The certainty of evidence, categorized as high, moderate, low, or very low, was estimated by evaluating risk of bias, inconsistency, imprecision, indirectness, and publication bias. The certainty rating for each outcome was downgraded when serious concerns were identified in any of these domains.

### 2.6 Statistical analysis

The meta‐analysis was conducted by two authors (ICP and EOG) using Comprehensive Meta‐Analysis version 4 (Biostat, Englewood, New York, USA). The pooled effect was calculated using the Cohen's standardized mean difference (SMD) with its 95% confidence interval (95% CI) under a random‐effects model.^36^, ^37^ Effects sizes were categorized as no effect (SMD 0), small (SMD 0.2–0.4), medium (SMD 0.4–0.7) or large (SMD >0.8).^38^ To further evaluate clinical relevance, when studies utilized the same instrument for a specific outcome, the analysis was complemented by calculating the minimal clinically important difference (MCID). The MCID was defined as *“*the smallest difference in score in the domain of interest, which patients perceive as beneficial and which would mandate, in the absence of troublesome side effects and excessive cost, a change in the patient*'*s management.”^39^ The results of the meta‐analyses are displayed using forest plots.^40^ The risk of publication bias was assessed through visual inspection of funnel plots, Egger's test, and Duval and Tweedie's trim‐and‐fill method.^41^, ^42^ The trim‐and‐fill method estimates an adjusted pooled effect size by accounting for potential publication bias.^43^ If the difference between the original and adjusted pooled effect size exceeded 10%, the certainty of evidence was downgraded by one level.^44^ Statistical heterogeneity was evaluated through the degree of inconsistency of Higgins (*I* ^2^) the *χ*‐square test and its *P* value,^45^ which was categorized as low (*I* ^2^ 5%–25%), moderate (*I* ^2^ 25%–50%) or high (*I* ^2^ >50%).^45^, ^46^ Subgroup analyses were performed based on two main criteria: the type of IVR software (passive vs. active) and the specific gynecologic procedure, requiring a minimum of two comparisons per subgroup.

## 3 RESULTS

### 3.1 Study selection

A total of 343 records were identified from electronic databases (*n* = 337) and other sources (*n* = 6). After removing 149 duplicates, 189 records were screened by title and abstract. Of these, 169 were excluded (167 for being irrelevant and 2 for failing to meet the PICOS criteria). Ultimately, 25 RCTs met the eligibility criteria and were included in the review (Figure 1).^47^, ^48^, ^49^, ^50^, ^51^, ^52^, ^53^, ^54^, ^55^, ^56^, ^57^, ^58^, ^59^, ^60^, ^61^, ^62^, ^63^, ^64^, ^65^, ^66^, ^67^, ^68^, ^69^, ^70^, ^71^ Inter‐rater agreement during this screening phase was excellent (*κ* = 0.88).

**FIGURE 1:**
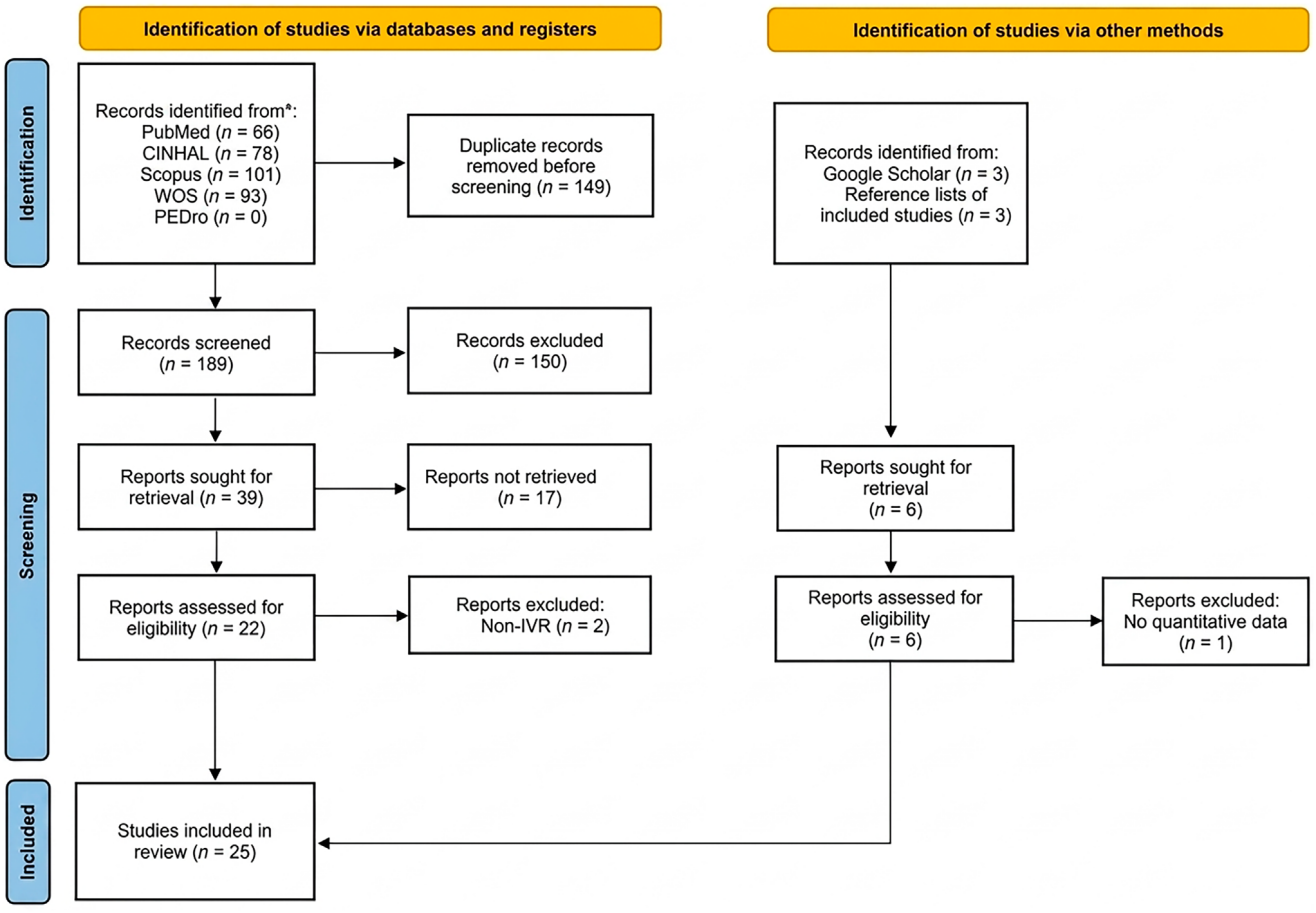
PRISMA flow diagram of the study selection process.

### 3.2 Characteristics of the studies included

The 25 included RCTs enrolled a total of 2438 women (mean age: 35.3 ± 12.6 years old) and were conducted between 2015 and 2025 across 12 countries: Spain,^52^, ^61^, ^63^ Turkey,^47^, ^48^, ^54^, ^62^, ^68^, ^69^ the USA,^50^, ^59^ France,^49^ the UK,^51^, ^67^ Israel,^53^, ^64^, ^70^ Egypt,^55^, ^60^ Germany,^56^ Iran,^57^, ^58^ Netherlands,^65^ Belgium,^66^ and Italy.^71^ The participants underwent various gynecologic procedures, including hysteroscopy,^50^, ^51^, ^52^, ^53^, ^63^, ^66^, ^67^, ^71^ hysterosalpingography,^47^, ^48^ episiotomy,^54^, ^55^, ^57^, ^58^, ^60^, ^62^, ^64^, ^68^ colposcopy,^56^ hysterectomy,^59^ intrauterine device (IUD) insertion,^49^ and transvaginal sonography.^70^ Participants in the intervention groups received IVR using various hardware and software setups (details in Table 1). A total of 21 RCTs employed passive visualization software,^47^, ^48^, ^49^, ^50^, ^51^, ^52^, ^54^, ^55^, ^56^, ^57^, ^58^, ^61^, ^62^, ^64^, ^65^, ^66^, ^67^, ^68^, ^69^, ^70^, ^71^ whereas four utilized active software.^53^, ^59^, ^60^, ^63^ Across all included studies, the intervention consisted of a single IVR session administered either during or immediately following the gynecologic procedure. Consequently, exposure time varied across trials, as the duration was inherently linked to the specific procedure being performed. Regarding the control groups, patients primarily received usual care, although some trials incorporated specific variations such as skin‐to‐skin contact,^68^ listening to nature sounds,^48^ or mobile‐assisted pain education.^47^ Pain was evaluated using the visual analog scale (VAS),^47^, ^48^, ^49^, ^50^, ^52^, ^54^, ^55^, ^56^, ^59^, ^60^, ^61^, ^62^, ^63^, ^64^, ^65^, ^66^, ^68^, ^69^ and the numeric rating scale (NRS).^51^, ^53^, ^57^, ^67^, ^70^, ^71^ Anxiety was assessed using the state–trait anxiety inventory (STAI),^48^, ^52^, ^56^, ^60^, ^64^, ^68^, ^69^, ^70^, ^71^ the NRS,^51^, ^58^, ^67^ and the VAS.^55^, ^66^ Finally, five RCTs reported receiving external funding.^47^, ^49^, ^51^, ^52^, ^61^ Comprehensive details regarding the study characteristics and IVR protocols are presented in Table 1.

**TABLE 1:** Characteristics of the studies included.

Study	Participants (*N*, procedure, age)	VR group	Control group	Outcomes	
		*N*, characteristics the VR‐based intervention	*N*, Control intervention characteristics	Variable (test) and time points	Qualitative findings
Bal and Kulakaç 2023^47^ (Turkey)	126 women undergoing hysterosalpingography	Forty women (27.4 ± 3.14 years) used 360° IVR video showing nature scenes during hysterosalpingography	Control group_1_: Forty patients were included in mobile‐assisted education group	Pain intensity (VAS)	No differences were found in pain scores during the HSG between the intervention group and the control groups (*P* > 0.05)
Non‐blinded RCT	Mean age: 27.9 ± 4.5	One session (duration of the HSG procedure). Passive visualization (360° nature scenes)	Control group_2_: Forty patients received usual care		Women who received routine care reported higher post‐intervention pain levels than those assigned to the RVI group or the education group
Setting: Turkey Hospital					
Funding: Yes (Scientific Research Projects Coordination Unit, Ondokuz Mayis University)					
Baltaci et al. 2024^48^ (Turkey)	135 women undergoing HSG	Forty‐five women (29.6 ± 6.1 years) used IVR with 360° nature video + nature sounds during HSG	Control group_1_: Forty‐five patients were included in nature sounds group	Pain (VAS)	Significant reduction in pain during HSG (*P* = 0.013). No difference between IVR + NS and NS groups (*P* > 0.05)
Single‐blind RCT		One session (45 min: 30 min before and 15 min during). Passive visualization (nature video with sounds)	Control group_2_: Forty‐five patients received usual care	Anxiety (STAI)	IVR + NS group showed lower pain than control (*P* = 0.009)
Setting: Hospital in Turkey					Significant differences in anxiety post‐HSG (*P* = 0.000)
Funding: None					
Benazzouz et al. 2024^54^ (France)	95 women scheduled for IUD insertion	Forty‐seven women (23.8 ± 4.9 years) were assigned to the intervention group	Forty‐eight women (23.5 ± 4.6 years) received standard care	Pain (VAS)	No statistically significant differences in pain levels between groups during IUD insertion (*P* = 0.54)
Non‐blinded RCT		Intervention: IVR during IUD insertion showing immersive landscapes such as forests, ocean floors, or outer space		Anxiety (VAS)	Anticipated pain levels before insertion were significantly correlated with perceived pain during the procedure (*P* = 0.0015)
Setting: Family Planning Center of Rennes and Abortion Center of Rennes University Hospital		One session (approximately 5 min). Passive visualization (landscapes)			Anxiety levels during IUD insertion were significantly correlated with pain levels during the procedure (*P* = 0.0021)
Funding: Yes (Institut de la Mère et de l'Enfant, Rennes and Rennes University Hospital)					
Brunn et al. 2022^50^ (USA)	50 women undergoing outpatient hysteroscopy	Twenty‐five women (37 ± 4.24 years) received IVR during hysteroscopy	Twenty‐five women (37 ± 5.18 years) received standard care	Pain (VAS)	No statistically significant differences in pain scores during or after hysteroscopy procedures between the IVR group and the control group (*P* > 0.05)
Non‐blinded RCT		Intervention: A meditation app + IVR was used. Device: Oculus GO		Adverse events	One case of blurred vision
Setting: Outpatient minimally invasive gynecologic surgery clinic		One session (duration of the office‐based hysteroscopy). Passive visualization (guided meditation app with calming music)			
Funding: Not reported					
Deo et al. 2021^51^ (UK)	40 women scheduled for outpatient hysteroscopy	Twenty women (31.1 ± 5.4 years) received guided relaxation through IVR	Twenty women (31.3 ± 5.2 years) received standard care	Pain (NRS)	Compared to standard care, the IVR intervention significantly reduced worst pain (7.85 vs. 5.65; *P* = 0.011)
Non‐blinded RCT		Device: Oculus GO			Mean pain was reduced in the IVR group by 2.3 points (6.0 vs. 3.7; *P* = 0.009)
Setting: UK University Hospital		One session (8 min). Passive visualization (guided relaxation)		Anxiety (NRS)	Anxiety reduced by 2.15 points in IVR group compared to control (5.45 vs. 3.3; *P* = 0.024)
Funding: Yes (NIHR Patient Safety Research Center)				Adverse effects	One case of nausea and one case of claustrophobia
Estadella‐Tarriel et al. 2025^52^ (Spain)	159 women undergoing outpatient hysteroscopy	Seventy‐nine women (43.3 ± 10 years) received IVR intervention “Under the Sea” 3D interactive distraction environment, asking patients to identify marine life	Eighty women (45.6 ± 9.9 years) received standard care	Pain (VAS)	Significantly lower pain in IVR group during hysteroscopy (4.51 vs. 5.59; *P* = 0.013)
Parallel‐group non‐blinded RCT		Device: PICO G2 VR headset			Lower pain 10 min post‐procedure in IVR group (2.09 vs. 3.33; *P* = 0.001)
Setting: Hospital Santa Creu i Sant Pau (Barcelona) and Hospital Arnau de Vilanova (Lleida)		One session (7 min). Passive visualization (guided relaxation “body‐scan”)		Anxiety (STAI‐T)	Among low‐anxiety patients, IVR group reported significantly less pain (*P* = 0.052), which remained 10 min after (*P* = 0.002)
Funding: Yes (Digital Health Validation Center, Sant Pau Hospital)					
Fouks et al. 2021^53^ (Israel)	82 women scheduled for hysteroscopy	Forty‐four women (mean age 39.0) assigned to IVR intervention (diving scenario)	Thirty‐eight women (mean age 38.0) received standard care	Pain (NRS)	No significant differences in pain scores (*P* = 0.67)
Single‐blind RCT		Device: SootheVR headset			IVR group reported lower postoperative pain (*P* = 0.04)
Setting: Tel Aviv University‐Affiliated Medical Center		One session (mean 8.1 min). Active interaction (diving scenario)			
Funding: No					
Gökduman‐Keles and Altinkaya 2025^54^ (Turkey)	84 pregnant women undergoing episiotomy	Forty women (22.3 ± 3.5 years) received IVR intervention (natural landscapes during episiotomy)	Forty‐four women (21.2 ± 2.7 years) received routine care	Pain (VAS)	IVR group reported significantly reduced perception of procedure duration (*P* < 0.001)
Single‐blind RCT		Device: Everest VR‐0023 3D Virtual Reality Glasses			Pain higher in control group during and 1‐h post‐episiotomy (*P* < 0.001)
Setting: Obstetrics and Gynecology Hospital, Eastern Mediterranean region		One session (approximately 15–20 min). Passive visualization (natural landscapes)			Pain increase significantly greater in control group (*P* < 0.001)
Funding: No					
Ahmed et al. 2022^55^ (Egypt)	200 primiparous women post‐episiotomy	One hundred women (21.05 ± 4.14 years) used IVR during postpartum	One hundred women (21.57 ± 4.60 years) received standard care Eighty‐three women (44.0 ± 7.3 years) received standard care	Pain (NRS)	Significant pain and anxiety reduction in IVR group at 60 min post‐intervention (*P* < 0.001)
Pilot RCT		One session (approximately 5 min). Passive visualization (immersive landscapes)			Fewer women with severe pain in IVR group (33%) versus control (70%)
Setting: General Qena Hospital and South‐Valley University Hospital postpartum department				Anxiety (VAS)	Severe anxiety in 10% of IVR group versus 30% in control
Funding: Not reported					
Hecken et al. 2023^56^ (Germany)	247 women undergoing colposcopy	Intervention group: Eighty‐two women (42.6 ± 5.6 years) received IVR before and during colposcopy		Pain (VAS)	No significant differences in pain during colposcopy (*P* = 0.65)
Non‐blinded RCT		Device: Pico G2 4K VR headset		Anxiety (STAI‐S)	Pain after procedure similar across groups (median of 10, *P* = 0.89)
Setting: Marien Herne Hospital		One session (10 min + duration of the colposcopy). Passive visualization (nature/underwater films and cartoons)			No significant differences in perceived anxiety (*P* = 0.44)
Funding: No		Intervention group_2_: Eighty‐two women (42.7 ± 6.2 years) received IVR before colposcopy			
		Device: Pico G2 4K VR headset			
		One session (10 min). Passive visualization (nature/underwater films and cartoons)			
Jahanishoorab et al. 2015^57^ (Iran)	30 pregnant women (24.1 ± 4.1 years) during episiotomy	Fifteen women received IVR Intervention: 3D film (dolphins and whales) through VR glasses	Fifteen women received standard care	Pain (NRS)	Significantly reduced pain in IVR group during procedure (16.7 vs. 39.3; *P* = 0.038)
Non‐blinded RCT		Device: Vuzix Wrap 920			Lower pain in hymen repair (9.0 vs. 23.6; *P* = 0.038)
Setting: Omolbanin Hospital		One session (mean 11.4 min). Passive visualization (3D film: Dolphins and whales)			
Funding: Not reported					
Shoorab et al. 2016^58^ (Iran)	30 pregnant women (24.1 ± 4.1 years) during episiotomy	Fifteen women received IVR Intervention: 3D film (dolphins and whales) through VR glasses	Fifteen women received standard care	Anxiety (NRS)	Significant reduction in anxiety during and after repair (*P* = 0.000)
Non‐blinded RCT		Device: Vuzix Wrap 920			No baseline differences between groups
Setting: Omolbanin Hospital		One session (duration of the episiotomy repair). Passive visualization (3D film: Dolphins and whales)			IVR was safe and effective for anxiety reduction
Funding: Not reported					
McCune et al. 2023^59^ (USA)	30 women post‐laparoscopic hysterectomy for benign conditions	Fifteen patients (41.7 ± 9.5 years) used VR postoperatively	Fifteen women (40.9 ± 14.3 years) received standard care	Pain (VAS)	No significant differences in postoperative pain (VR: 5.5 vs. control: 6.1; *P* = 0.39)
Non‐blinded RCT	One session (mean 27.4 min). Mixed software (patient choice between passive visualization of nature sceneries/meditations and active interaction with “Merry Snowballs” game)				Similar opioid use (VR: 32.8 mg vs. control: 31.8 mg morphine equivalent; *P* = 0.88)
Setting: Banner University Medical Center, Phoenix, AZ					
Funding: Not reported					
Mohamed‐Nabil Aboushady et al. 2023^60^ (Egypt)	100 primiparous women during delivery	Fifty women (28.86 ± 5.96 years) received IVR during episiotomy repair	Fifty women (28.52 ± 6.21 years) received standard care	Pain (VAS)	Shorter repair time in IVR group (9.72 ± 3.15 min vs. 15.10 ± 4.25 min; *P* < 0.05)
Non‐blinded RCT		Intervention: Ocean scenes + local anesthesia		Anxiety (STAI‐S)	Less pain during repair (4.66 ± 1.61 vs. 6.82 ± 1.00; *P* < 0.05)
Setting: Menoufia University Hospital		On we session (mean 9.72 min). Active interaction (ocean scene with marine mammals where patients controlled the view and took underwater pictures using a hand controller)			Less anxiety and higher maternal satisfaction during procedure (*P* < 0.05)
Funding: Not reported					
Olloqui et al. 2025^61^ (Spain)	120 women scheduled for outpatient hysteroscopy	Sixty women (44.20 ± 12.95 years) received IVR	Sixty women (46.23 ± 10.66 years) received standard care	Pain (VAS)	No differences in perceived pain or satisfaction (*P* > 0.05)
Unblinded RCT		Device: Oculus Quest 2			No differences in mean intensity during the procedure (*P* > 0.05)
Setting: 12 de Octubre University Hospital, Madrid		One session (mean 4.57 min). Passive visualization (360° video of a gondola ride in Venice with relaxing music)			Intervention: 360° video of a gondola ride in Venice with relaxing music
Funding: Yes (Hysteroscopy and Minimally Invasive Surgery Unit)					
Orhan and Bülez 2023^62^ (Turkey)	50 pregnant women undergoing episiotomy	Twenty‐five women (21.4 ± 4.15) received IVR with anesthesia	Twenty‐five women (19.6 ± 2.63) received standard care	Pain (VAS)	Lower pain in IVR group during internal suturing (*P* = 0.014) and skin repair (*P* = 0.001)
Single‐blind RCT		Intervention: immersive relaxing videos (nature or underwater) during repair + lidocaine 5 mL			Higher satisfaction in IVR group (*P* = 0.001)
Setting: Kahramanmaras Necip Fazıl City Hospital Maternity Clinic		One session (mean 8.6 min). Passive visualization (underwater or nature videos with music)			
Funding: Not reported					
Pelazas‐Hernández et al.^63^ 2023 (Spain)	154 women scheduled for surgical hysteroscopy	Eighty‐two women (47.19 ± 8.72 years) received IVR.	Seventy‐two women (49.2 ± 11.79 years) received standard care	Pain (VAS)	Significantly lower pain during procedure in IVR group (VAS 2.45) vs. control (VAS 3.97) (*P* = 0.006)
Single‐blind RCT		Intervention: immersive experience “A Night Sky” during hysteroscopy			Lower pain 15 min post‐procedure in IVR group (VAS 1.77 vs. 3.30; *P* = 0.004)
Setting: El Escorial University Hospital		Device: Oculus GO			
Funding: No		One session (duration of the hysteroscopy). Active interaction (connecting constellations using a controller)			
Peleg et al. 2024^64^ (Israel)	88 pregnant women undergoing episiotomy or perineal tear repair	Forty‐four women (28.9 ± 3.9) used IVR	Forty‐four women (29.4 ± 5.1) received standard care	Pain (VAS), Anxiety (STAI)	Lower pain in IVR group (*P* < 0.005)
Non‐blinded RCT		One session (duration of the repair). Software type not specified			No differences in anxiety (*P* > 0.005)
Setting: Ziv Medical Center					
Funding: Not reported					
Rosielle et al. 2024^65^ (Netherlands)	134 women scheduled for HSG	Seventy‐nine women underwent HSG with IVR (nature films and breathing exercises during procedure)	Seventy‐five women received standard care	Pain (VAS)	No differences in maximum pain (VR 6.8 vs. control 6.6; *P* = 0.52) or overall pain (VR 5.0 vs. control 4.9; *P* = 0.88)
Non‐blinded RCT		Device: PICO G2 4K			Adjusting for expectations, VR group reported more pain (*P* < 0.05)
Setting: Amsterdam UMC		Software: SyncVR Relax & Distract			Satisfaction similar, though women in VR group preferred repeating with VR
Funding: Not reported		One session (duration of the HSG procedure). Passive visualization (relaxing movies and breathing exercises)			
Schutyser et al. 2021^66^ (Belgium)	48 women undergoing hysteroscopy or endometrial biopsy	Twenty‐five women (21.4 ± 4.15 years) assigned to IVR	Twenty‐three women received standard care	Pain (VAS)	No significant differences in pain or anxiety before, during, or after the procedure (*P* > 0.05)
Non‐blinded RCT		Intervention: relaxation with self‐hypnosis (images + sounds) using VR glasses with headphones		Anxiety (VAS)	Pain increased during and decreased after in both groups, with no differences
Setting: Brussels University Hospital		Software: Oncomfort			Average procedure time similar (*P* = 0.09)
Funding: Not reported		One session (mean 3 min 43 s procedure +2 min induction). Passive visualization (autohypnosis relaxation program with image and sound distraction)			
Sewell et al. 2023^67^ (UK)	83 women scheduled for outpatient hysteroscopy	Forty‐one women (47.8 ± 11.3 years) received IVR intervention (Japanese zen garden with relaxing music + guided breathing)	Forty‐two women (49.6 ± 11.3 years) received standard care	Pain (NRS)	Significantly lower anxiety in IVR group (*P* = 0.03)
Non‐blinded RCT		Device: Pico G2.		Anxiety (NRS)	No significant differences in mean pain (*P* = 0.41) or maximum pain (*P* = 0.71)
Setting: University Hospital of London		Software: HypnoVR			No differences in procedure duration or recovery room time
Funding: Not reported		One session (mean 26 min in procedure room). Passive visualization (Japanese zen garden with music and breathing exercises)		Adverse effects	VR group: 2/41 (4.87%)
Şolt Kırca et al. 2023^68^ (Turkey)	120 primiparous women undergoing episiotomy repair	Forty women (23.52 ± 3.41 years) received IVR during episiotomy	Control group_1_: Forty women (22.57 ± 4.11 years) received standard care	Pain (VAS)	Mean pain: 1.8 (VR), 4.69 (skin‐to‐skin), 4.7 (control) (*P* < 0.001)
Double‐blind RCT		One session (duration of the episiotomy repair). Passive visualization (software content not specified in this section)	Control group_2_: Forty women received skin‐to‐skin contact during episiotomy	Anxiety (STAI)	Mean anxiety: 53.47 (VR), 55.5 (skin‐to‐skin), 55.65 (control) (*P* < 0.05)
Setting: Hospital in Turkey					
Funding: Not reported					
Yılmaz Sezer et al. 2023^69^ (Turkey)	62 women with primary infertility undergoing hysterosalpingography	Thirty‐one women (30.4 ± 6.4 years) received RVI during hysterosalpingography.	Thirty‐one women (31.5 ± 5.6 years) received standard care	Pain (VAS)	Non‐significant differences in the mean levels of pain (*P* = 0.655) and anxiety (*P* = 0.114)
Non‐blinded RCT		Device: VR SHINECON Headset		Anxiety (SAI)	Significant differences between worst pain scores during (*P* = 0.000) and 15 min after (*P* = 0.015) hysterosalpingography
Setting: Ankara University Hospital		One session (5–10 min). Passive visualization (360‐degree videos of sea, underwater, and forest with relaxing music)			
Funding: No					
Zajicek et al. 2024^70^ (Israel)	55 women undergoing TVS for endometriosis	Thirty‐two women with endometriosis were assigned to VR‐assisted transvaginal sonography	Twenty‐three women were assigned to conventional transvaginal sonography	Pain (NRS)	No significant differences were found in baseline pain (*P* = 0.19) or STAI‐s scores (*P* = 0.90). Pain scores after the intervention (*P* = 0.03) and post‐intervention anxiety levels (*P* = 0.04) were significantly lower in the VR group
Non‐blinded RCT		One session (duration of the TVS procedure). Passive visualization (software content not specified)		Anxiety (STAI‐s)	
Setting: single tertiary center					
Funding: Not reported					
Zizolfi et al. 2025^71^ (Italy)	116 women scheduled for outpatient hysteroscopy	Fifty‐eight women (45.0 ± 13.00 years) were allocated to VR group	Fifty‐eight women (43.24 ± 11.26 years) received standard outpatient hysteroscopy	Pain (NRS)	Post‐procedure pain scores were significantly lower in the VR group compared to controls (*P* = 0.005). Reductions in pain from pre‐ to post‐procedure were greater in the VR group (*P* < 0.0001). Post‐procedure anxiety scores were also significantly lower in the VR group (*P* = 0.0006), and reductions in anxiety from pre‐ to post‐procedure were greater in the VR group (*P* < 0.0001)
Non‐blinded RCT		Device: Deepsen VRx with hypno VR software		Anxiety (NRS)	
Setting: Hysteroscopy Unit of University of Naples “Federico II”		One session (mean 6.94 min). Passive visualization (hypno VR software for clinical hypnosis and relaxation)		Adverse effects	VR group: 2/58 (3.45%)
Funding: No					Control group: 1/58 (1.72%)

Abbreviations: HSG, hysterosalpingography; IUD, intrauterine device; IVR, immersive virtual reality; NRS, numerical rating scale; NS, nature sounds; RCT, randomized controlled trial; STAI, state–trait anxiety inventory; TVS, transvaginal sonography; VAS, visual analog scale; VR, virtual reality.

### 3.3 Assessment of the risk of bias in individual RCTs

Although all included studies were RCTs with adequate random sequence generation, appropriate allocation concealment was lacking in 21 trials (84%), indicating a potential risk of selection bias. Furthermore, due to the nature of the experimental intervention (IVR), the blinding of participants and personnel was not feasible in any study, resulting in a high risk of performance bias across all trials (100%). Similarly, outcome assessors were not blinded in any of the RCTs, introducing a high risk of detection bias. Conversely, the risk of attrition, reporting, and other biases was low across all studies. The RoB assessment for each individual trial is detailed in Figure 2, while the overall risk of bias summary is presented in Figure S1.

**FIGURE 2:**
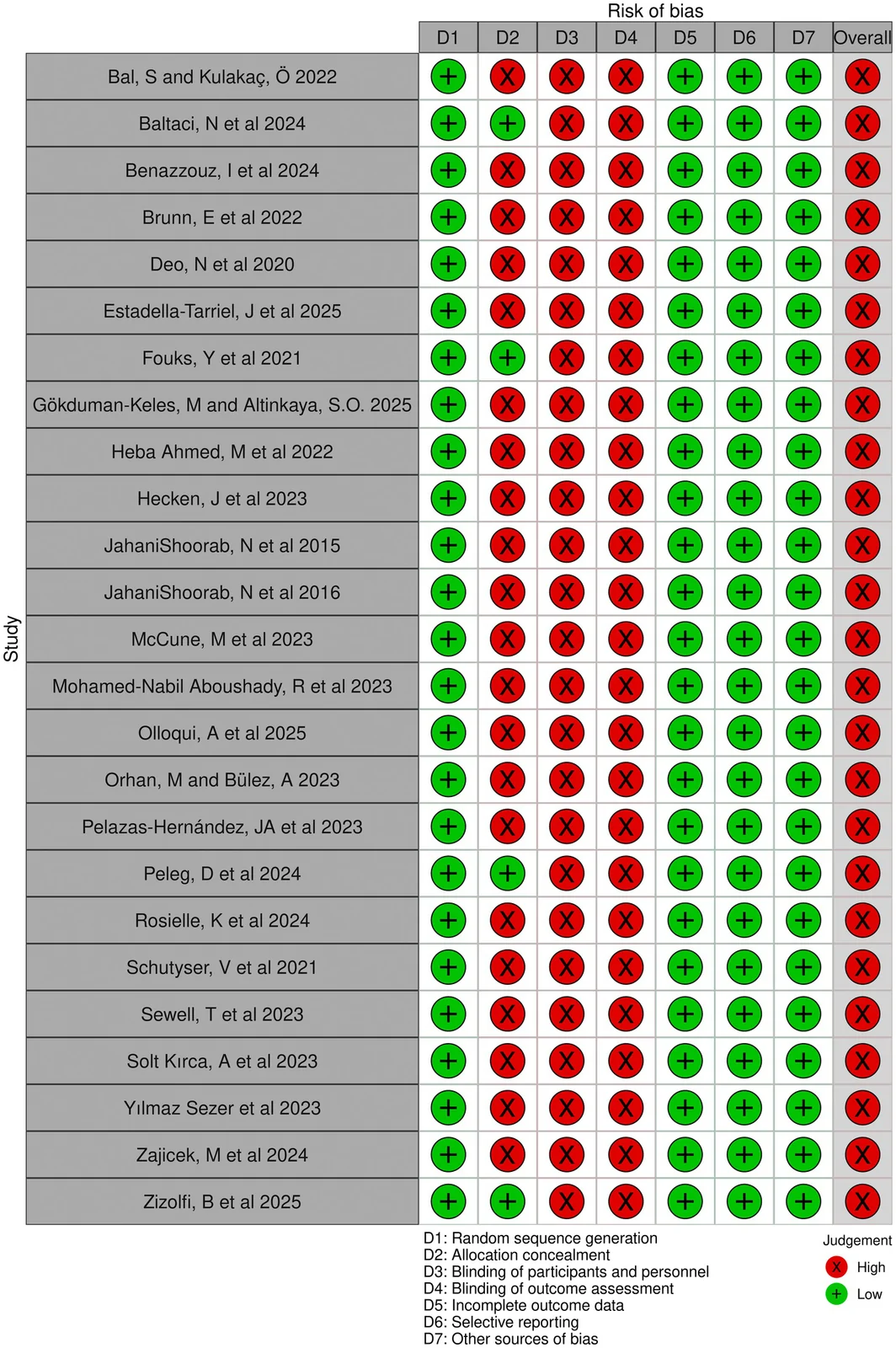
Cochrane Risk of Bias Tool assessment for each randomized controlled trial (RCT) included.

### 3.4 Quantitative findings

#### 3.4.1 Efficacy of IVR in reducing pain intensity during and after the intervention

A total of 17 studies^47^, ^48^, ^49^, ^50^, ^51^, ^52^, ^53^, ^54^, ^56^, ^57^, ^60^, ^61^, ^62^, ^65^, ^67^, ^68^, ^69^ with 19 independent comparisons reported data from 1801 women (94.8 per comparison). The results showed, with moderate quality of evidence, a medium effect (SMD = −0.46; 95% CI: −0.71 to −0.21; *P* < 0.001; *I* ^2^ = 12.7%; *χ* ^2^ = 20.6; degree of freedom [df] = 18; *P* = 0.3) of IVR in reducing pain intensity during gynecologic procedures (Figure 3a, Table 2), without risk of publication bias (Egger's test *P* = 0.1). Additionally, this meta‐analysis reported that IVR was able to reduce pain intensity 1.2 points (95% CI: −1.78 to −0.53; *P* < 0.001; *I* ^2^ = 25.7%; *χ* ^2^ = 24.2; df = 18; *P* = 0.14). Subgroup analysis reported a medium effect using passive IVR (SMD = −0.41; 95% CI: −0.64 to −0.17; *P* = 0.001), and non‐statistically significant differences using the active IVR software (SMD = −0.81; 95% CI: −2.40 to −0.77; *P* = 0.78). Second subgroup analysis reported effectiveness of IVR during episiotomy (SMD = −1.11; 95% CI: −1.16 to −0.61; *P* < 0.001), hysterosalpingography (SMD = −0.27; 95% CI: −0.44 to −0.10; *P* = 0.003) and hysteroscopy (SMD = −0.21; 95% CI: −0.38 to −0.04; *P* = 0.016).

**FIGURE 3:**
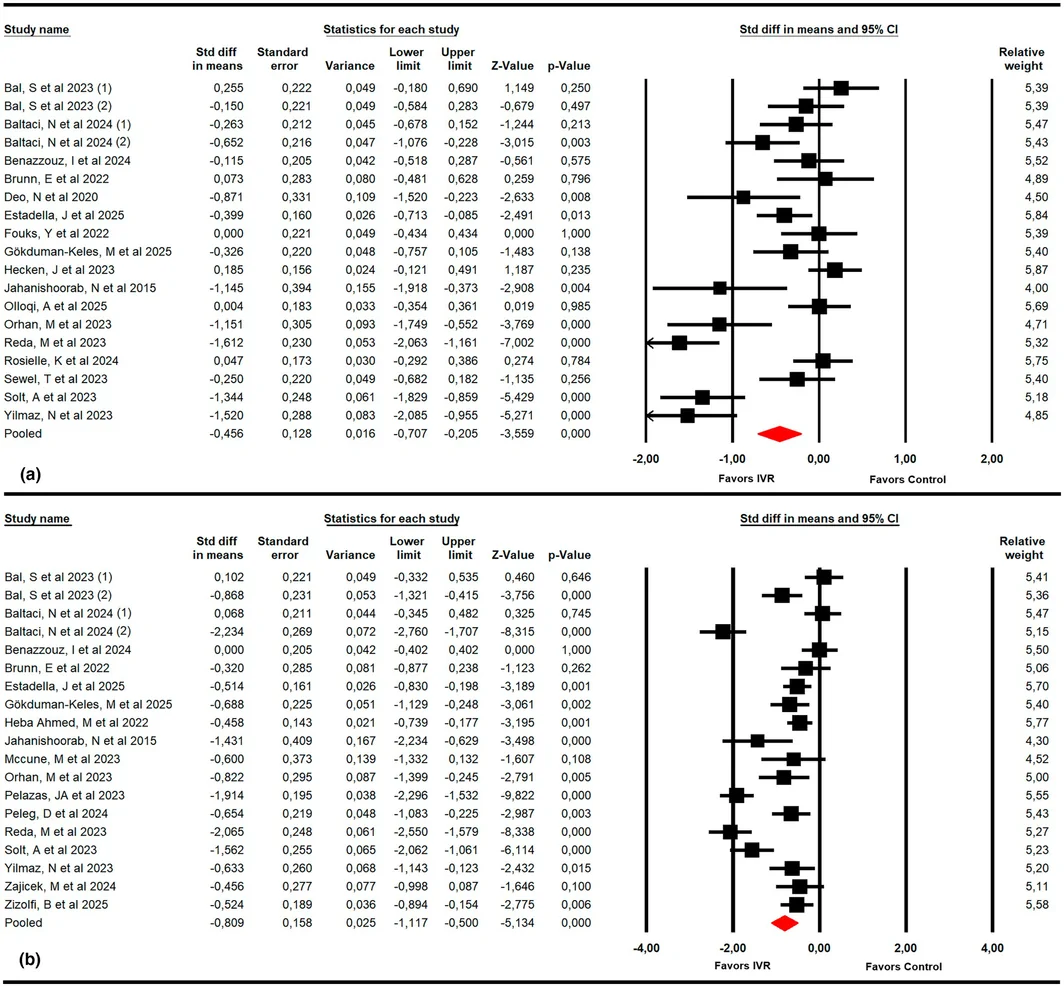
Forest plot for the effectiveness of immersive virtual reality (IVR) in reducing peri‐ and post‐procedural pain.

**TABLE 2:** Main findings in meta‐analyses.

Outcome	Time point	Findings summary											Quality evidence (GRADE)					
		Effect size						Heterogeneity		Publication bias								
		*K*	*N*	N_s_	SMD	95% CI	*P*	*Q* (df)	*I* ^2^ *(P)*	Egger *P*	Trim and fill		Risk of bias	Incons	Indir	Impr	Pub bias	Evidence strength
											Adj SMD	% Var						
Pain	During	19	1801	94.8	−0.46	(−0.71 to −0.21)	<0.001	20.6 (18)	12.7 (0.3)	0.1	−0.46	0	Moderate	No	No	No	No	Moderate
	After	19	1820	95.8	−0.82	(−1.12 to −0.5)	<0.001	18.7 (18)	4.1% (0.41)	0.27	−0.82	0	Moderate	No	No	No	No	Moderate
Anxiety	During	8	649	81.1	−0.33	(−0.63 to −0.03)	0.031	9.3 (7)	24.9 (0.23)	0.46	−0.25	25	Moderate	No	Yes	No	Yes	Low
	After	11	1036	94.2	−0.76	(−1.22 to −0. 3)	0.001	12.6 (10)	19.4 (0.25)	0.62	−0.76	0	Moderate	No	Yes	No	No	Low

Abbreviations: Adj, adjusted; CI, confidence Interval; df, degree of freedom; GRADE, grades of recommendation, assessment, development; *I* ^2^, degree of inconsistency of Higgins; Impr, imprecision; Incons, inconsistency; Indir, indirectness; *K*, number of studies (comparisons) per meta‐analysis; *N*, number of participants included in each meta‐analysis; Ns, average number of participants per meta‐analysis; *P*, *P* value; Pub, publication; *Q*, *Q*‐test; SMD, Cohen's standardized mean difference; % var., percentage of variation.

Second, 17 studies^47^, ^48^, ^49^, ^50^, ^52^, ^54^, ^55^, ^57^, ^60^, ^62^, ^63^, ^64^, ^68^, ^69^, ^70^, ^71^ comprising 19 independent comparisons provided data from 1820 women (95.8 per comparison). The meta‐analysis reported a large effect with moderate quality of evidence (SMD = −0.82; 95% CI: −1.12 to −0.5; *P* < 0.001; *I* ^2^ = 4.1%; *Q* = 18.7; df = 18; *P* = 0.41) in favor of IVR for relieving pain post‐intervention (Figure 3b and Table 2), without risk of publication bias (Egger's test *P* = 0.27). Additionally, this meta‐analysis reported that IVR was able to reduce pain intensity 1.6 points (95% CI: −2.13 to −1.1; *P* < 0.001; *I* ^2^ = 39.3%; *χ* ^2^ = 29.7; df = 18; *P* = 0.04). Subgroup analysis reported that passive (SMD = −0.66; 95% CI: −0.95 to −0.37; *P* < 0.001), and active IVR software (SMD = −1.6; 95% CI: −2.4 to −0.87; *P* < 0.001) are effective in reducing pain post‐gynecologic procedure. Second subgroup analysis reported effectiveness of IVR during episiotomy (SMD = −0.9; 95% CI: −1.12 to −0.77; *P* < 0.001), hysterosalpingography (SMD = −0.58; 95% CI: −0.79 to −0.38; *P* < 0.001) and hysteroscopy (SMD = −0.84; 95% CI: −1 to −0.65; *P* < 0.001).

#### 3.4.2 Effectiveness of IVR in reducing anxiety levels during and after the intervention

Eight studies^49^, ^51^, ^56^, ^58^, ^60^, ^64^, ^66^, ^67^ provided eight independent comparisons and data from 649 women (81.1 per comparison). The overall meta‐analysis showed a small effect with low quality of evidence (SMD = −0.33; 95% CI: −0.63 to −0.03; *P* = 0.031; *I* ^2^ = 24.9%; *Q* = 9.3; df = 7; *P* = 0.23) in favor of IVR in reducing anxiety during the gynecologic procedures (Figure 4a, Table 2). There was evidence of potential publication bias (Egger's test *P* = 0.06). Consequently, the trim‐and‐fill method was applied, indicating that this potential bias may have overestimated the original pooled effect size of IVR by approximately 25% (adjusted SMD = −0.25) (Figure S2). Subgroup analyses based on IVR software type could not be performed, as the active IVR subgroup comprised only a single comparison. However, a second subgroup analysis by procedure type demonstrated the effectiveness of IVR in reducing anxiety during episiotomy (SMD = −0.86; 95% CI: −1.38 to −0.35; *P* = 0.001).

**FIGURE 4:**
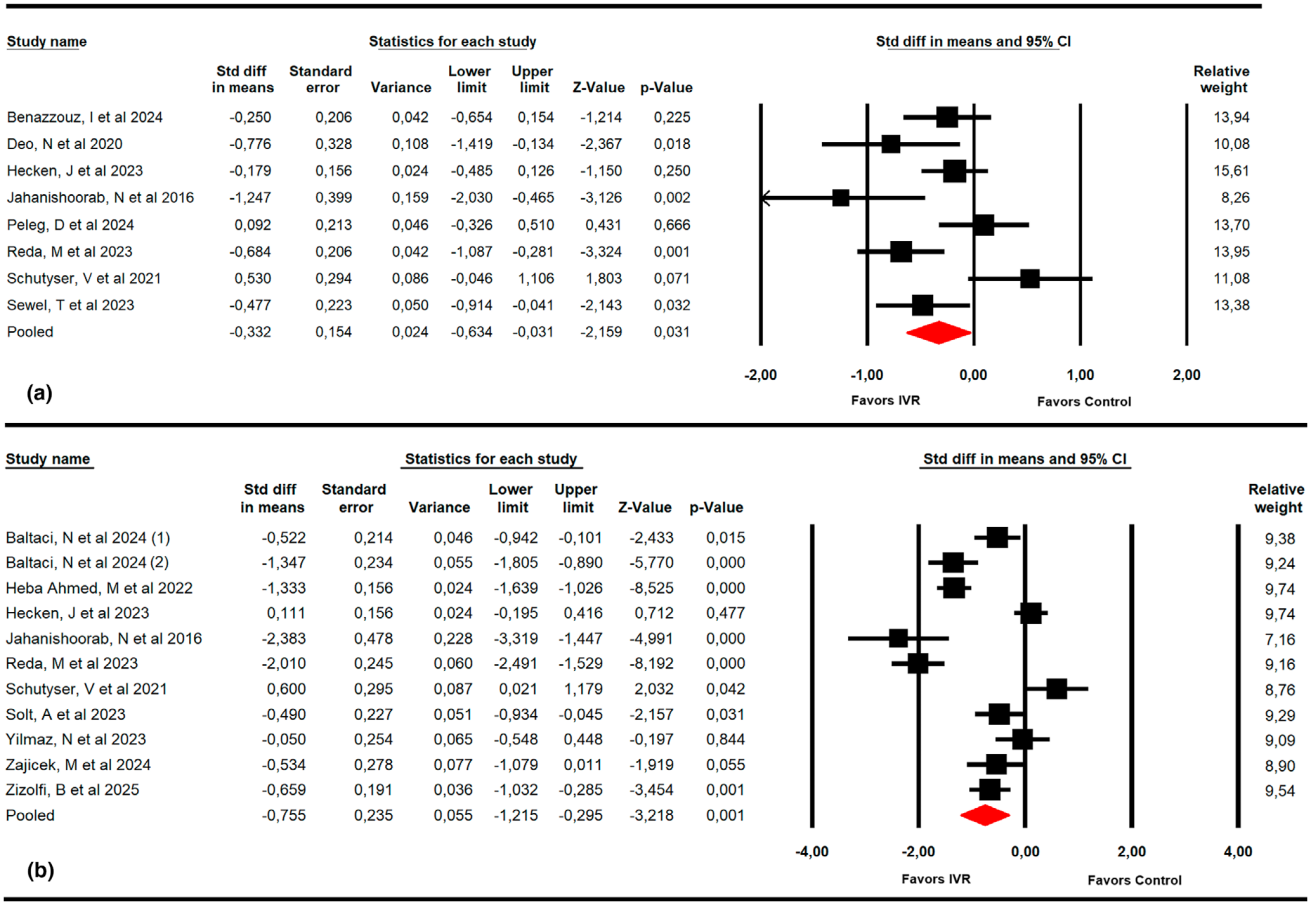
Forest plot for the effectiveness of immersive virtual reality (IVR) in reducing peri‐ and post‐procedural anxiety.

A total of 10 studies^48^, ^55^, ^56^, ^58^, ^60^, ^66^, ^68^, ^69^, ^70^, ^71^ with 11 independent comparisons reported data from 1036 women (94.2 per comparison). The meta‐analysis showed medium‐large effect (SMD = −0.76; 95% CI: −1.22 to −0.30; *P* = 0.001; *I* ^2^ = 19.4%; *Q* = 12.6; df = 10; *P* = 0.25) with low quality of evidence in favor of IVR for reducing anxiety after the intervention (Figure 4b and Table 2), with no risk of publication bias (Egger's test *P* = 0.62). Subgroup analysis reported that passive (SMD = −0.62; 95% CI: −2.5 to −1.5; *P* < 0.001), and active IVR software (SMD = −0.41; 95% CI: −0.64 to −0.17; *P* = 0.001) are effective in reducing anxiety post‐gynecologic procedure. Second subgroup analysis reported effectiveness of IVR during episiotomy (SMD = −1.5; 95% CI: −2.2 to −0.8; *P* < 0.001), and hysterosalpingography (SMD = −0.66; 95% CI: −0.93 to −0.4; *P* < 0.001).

### 3.5 Qualitative findings: Adverse events

Adverse events associated with the IVR intervention were reported in only four studies.^50^, ^51^, ^67^, ^71^ Deo et al.^51^ described one patient who experienced nausea but completed the procedure using the IVR device, alongside another patient with a known history of claustrophobia who removed the headset at the beginning of the session. Brunn et al.^50^ reported a single case of blurred vision that resolved spontaneously following the discontinuation of the headset. Finally, Sewell et al.^67^ and Zizolfi et al.^71^ each observed mild nausea in two participants; in all cases, the symptoms were self‐limiting and did not require further medical intervention.

## 4 DISCUSSION

Based on a comprehensive synthesis of 25 RCTs^47^, ^48^, ^49^, ^50^, ^51^, ^52^, ^53^, ^54^, ^55^, ^56^, ^57^, ^58^, ^59^, ^60^, ^61^, ^62^, ^63^, ^64^, ^65^, ^66^, ^67^, ^68^, ^69^, ^70^, ^71^ enrolling 2438 women, this study provides a meaningful contribution to the field by demonstrating that IVR can be effective as an adjunctive, non‐pharmacological intervention for alleviating peri‐ and post‐procedural pain and anxiety in gynecologic settings. Furthermore, our analysis revealed that IVR reduced pain intensity by 1.2 and 1.6 points during and after gynecologic procedures, respectively. It is important to note that these findings cannot be directly compared to the MCID thresholds established by Farrar et al.^72^ and Salaffi et al.^73^ for chronic musculoskeletal pain, or by Gerlinger et al. for endometriosis‐associated pelvic pain.^74^ To definitively assert the clinical relevance of IVR for this specific population, comparisons should ideally be drawn against studies evaluating MCID in acute post‐procedural or post‐surgical settings. Nevertheless, a decrease of approximately 1.5 points in pain scores is widely recognized in literature as a clinically meaningful improvement. Concerning safety outcomes, only four of the 25 included RCTs^50^, ^51^, ^67^, ^71^ provided safety data. Although these studies reported a very low incidence of mild and transient events (such as blurred vision and nausea), this widespread underreporting across the remaining trials suggests that the safety profile of IVR remains incompletely characterized. Consequently, definitively asserting the absolute safety of this intervention would be premature. While IVR appears well‐tolerated based on the available evidence, future trials must incorporate standardized protocols for adverse event reporting to rigorously evaluate its safety.

In the ongoing search for effective non‐pharmacological interventions, our SRMA demonstrates that IVR reduces both pain and anxiety across the assessed time points, supported by moderate and low certainty of evidence, respectively. These findings underscore the potential of IVR as an innovative, patient‐centered approach. Subgroup analyses reported that post‐procedural pain can be alleviated using active IVR software, while for anxiety, passive IVR provided major effect. However, these findings are considered with caution, due to the low number of studies reporting active IVR software. Our results are consistent with the findings of the SRMA by Baradwan et al.^22^ which concluded that IVR is an effective tool for mitigating intra‐procedural pain and anxiety, as well as postoperative pain, during outpatient hysteroscopies (although no effects on post‐intervention anxiety were observed). In contrast, Cohen et al.^23^ concluded that while IVR could be implemented to reduce anxiety during hysteroscopies, it did not significantly contribute to pain reduction. Vitagliano et al.^24^ suggested that active IVR could be associated with a reduction in pain during outpatient hysteroscopies. Finally, Baradwan et al.^26^ and Manouchehri et al.^25^ found clinically and statistically significant reductions in both pain and anxiety, during and following the procedure, aligning closely with our findings. Our subgroup analyses suggest that IVR are effective in reducing peri‐ and post‐procedural pain during hysteroscopy, episiotomy and hysterosalpingography. Concerning adverse events, Cohen et al.^23^ reported safety profiles like ours, further supporting the apparent safety and tolerability of this tool. In contrast to these previous SRMAs, which were constrained by small sample sizes and the pooling of heterogeneous study designs, the present study is the first SRMA to demonstrate, encompassing the largest cohort of women and strictly limiting inclusion to RCTs to date, that IVR devices are a useful adjunctive tool for pain and anxiety reduction across a broad spectrum of gynecologic procedures, including hysteroscopy, hysterosalpingography, episiotomies, colposcopy, hysterectomy, IUD insertion, and transvaginal sonography.

The modulation of pain perception using IVR devices occurs via stimulation of visual and sensorimotor networks.^75^ It is proposed that IVR provides patients with access to virtual experiences characterized by a profound sense of presence and that they engage the same neural circuits as embodied experiences, allowing the person to feel as if inhabiting a virtual body seen from a first‐person perspective.^76^, ^77^ Consequently, these interventions could evoke internal processes and neural pathways comparable to those underlying immersive experiences in individuals.^78^

IVR interventions may positively impact psychological well‐being by facilitating adaptation to the clinical environment, either by reducing the salience of acute stressors or by altering emotional and behavioral responses to such stimuli. Attentional distraction has been the primary mechanism proposed for acute pain reduction through IVR, and by generating such distraction, IVR can mitigate anticipatory anxiety and emotional reactivity to the procedure.^76^, ^79^ Moreover, it can modify individuals' responses to painful stimuli by affecting nociceptive neural signals, potentially leading to reductions in stress hormones and cortical activity related to pain perception.^80^ The findings of this meta‐analysis support these hypotheses, corroborating its effectiveness in managing pain and anxiety in the perioperative and postoperative stages.

The implementation of IVR as a non‐pharmacological tool to manage pain and anxiety during gynecologic procedures offers clear advantages, as IVR models are affordable and user‐friendly, facilitating their integration as complementary tools in gynecologic interventions and providing accessible solutions for patients. Although we previously noted that IVR is affordable and user‐friendly, its routine clinical implementation presents specific logistical challenges that must be considered. While standalone VR headsets are cost‐effective relative to conventional medical equipment, the upfront investment in hardware and specialized software subscriptions remains a significant consideration. Moreover, although the technology is highly intuitive for patients, its implementation requires dedicated staff training to operate the devices, resolve technical issues, and ensure seamless integration into fast‐paced clinical environments. Finally, the shared use of these headsets necessitates stringent infection control protocols. Facilities must enforce rigorous between‐patient hygiene measures to prevent cross‐contamination, including the use of disposable silicone covers, medical‐grade antimicrobial wipes, or ultraviolet‐C sterilization systems.^81^, ^82^ Despite its potential as a cost‐effective and feasible non‐pharmacological intervention, the integration of IVR into routine clinical practice faces implementation challenges that warrant consideration. These include restricted access to specialized hardware, the requirement for structured staff training, and potential barriers regarding patient acceptance. However, when situated within a multimodal framework, IVR serves as an effective strategy for managing pain and anxiety across diverse gynecologic settings. Beyond its effectiveness, IVR emerges as a safe and well‐tolerated adjunct to traditional clinical care. Our findings reinforce the expanding role of immersive technologies in perioperative medicine and underscore the need for future research aimed at the standardized integration of these tools into established clinical pathways.

The findings of this SRMA should, however, be interpreted in light of certain methodological limitations. First, the low number of RCTs included in the anxiety meta‐analysis may reduce the generalizability and level of evidence of this finding. Second, it is important to remark on limitations related to risk of bias in the RCTs included. Selection bias, identified in 21 RCTs due to inadequate allocation concealment, may have led to an overestimation of the findings, suggesting that the true magnitude of clinical efficacy should be interpreted with caution.^83^ Furthermore, the intrinsic nature of IVR interventions precludes the blinding of patients and clinicians, introducing performance bias potentially driven by the novelty effect and heightened participant expectations. The lack of blinded outcome assessors in these trials further introduces detection bias, a critical concern when evaluating subjective variables such as pain and anxiety. Collectively, these factors may inflate the observed treatment effect size.^84^, ^85^ Furthermore, publication bias was detected in the assessment of anxiety during gynecologic procedures, which overestimated the true effect of IVR by 25%, indicating that this finding can be considered with caution. Although this bias was corrected using the trim‐and‐fill method, it can be mentioned as an important limitation, due to reduce the generalization and soundness of this finding. However, it is generally recommended to include at least 10 studies to confidently assess publication bias, the low number of RCTs included in these assessments can reduce the precision of publication bias analyses. While all RCTs conducted a single session of IVR, the heterogeneity in software, video games, and IVR headsets should also be considered. A final limitation is that the heterogeneity of the assessment instruments used across studies precluded the calculation of the MCID for anxiety. Future studies should consolidate these results by ensuring better bias control, advocating for the standardization of IVR protocols, outcome measures, and standardized adverse event reporting, and recruiting appropriately large sample sizes.

## 5 CONCLUSION

This SRMA suggests that IVR is a promising, complementary non‐pharmacological approach that may alleviating pain and anxiety during and after gynecologic procedures in these women. Specifically, our findings elucidate a potential reduction in pain and anxiety levels, particularly after the gynecologic procedure. However, the effectiveness of IVR on reducing anxiety during gynecologic procedure can be considered with caution due to the detected publication bias advertisement. The prevalence of adverse events was low, suggesting that IVR seems to be a safe therapeutic option. These results show significant consistency, supported by low‐to‐moderate quality of evidence. The adoption of IVR represents a safe and well‐tolerated complementary adjunct with the potential to enhance conventional strategies for managing pain and anxiety in the gynecologic context. These findings underscore the expanding role of immersive technologies in perioperative medicine and highlight the need for future investigations directed toward their systematic and standardized integration into clinical work flows.

## AUTHOR CONTRIBUTIONS

CGL, MPL, ICP, HGL and EOG conceived and designed the study. CGL, ICP and EOG performed the literature search, study selection and data extraction. MPL and HGL contributed to quality assessment. ICP and EOG provided clinical expertise and contributed to the interpretation of findings. EOG supervised the statistical analyses. CGL, MPL, ICP, HGL and EOG drafted the manuscript. All authors critically revised the work for important intellectual content and approved the final version of the manuscript.

## FUNDING INFORMATION

The authors have nothing to report.

## CONFLICT OF INTEREST STATEMENT

The authors declare no conflict of interest.

## Supporting information


Table S1.

Data S1.

## Data Availability

Data is available from the authors upon reasonable request.
