# Supplementary material for: Effectiveness of immersive virtual reality‐based interventions for alleviating peri‐ and post‐procedural pain and anxiety in gynecologic settings: A systematic review and meta‐analysis of randomized controlled trials
Source: Int J Gynaecol Obstet. 2026 May 25;175(2):565–83. doi: 10.1002/ijgo.71095 (PMC13674823; doi:10.1002/ijgo.71095)
Supplement: Supplementary file 2 [file IJGO-175-565-s002.docx]

**Table S1.** Literature search strategies

| **Databases** | **Search strategy** |
| --- | --- |
| **PubMed Medline** | (Virtual Reality[mh] OR Virtual Reality Exposure Therapy[mh] OR Exergaming[mh] OR Virtual Reality[tiab] OR Virtual Reality Exposure Therapy[tiab] OR Virtual Reality Immersion Therapy[tiab] OR immersive virtual reality[tiab] OR exergam*[tiab] OR non-immersive virtual reality[tiab]) AND (Episiotomy[mh] OR Embryo Transfer[mh] OR Hysteroscopy[mh] OR colpotomy[mh] OR Episiotom*[tiab] OR Embryo Transfer*[tiab] OR Hysteros*[tiab] OR Uterine Endoscop*[tiab] OR Uteroscop*[tiab] OR Colposcop*[tiab] OR Culdosco*[tiab]) |
| **SCOPUS** | ( TITLE-ABS-KEY ( "Virtual Reality" OR "Virtual Reality Exposure Therapy" OR "Virtual Reality Immersion Therapy" OR "exergaming" OR "immersive virtual reality" ) AND TITLE-ABS-KEY ( "Colposcopy" OR "Culdoscopy" OR "Hysterosalpingography" OR "Hysteroscopy" OR “Episiotomy” ) ) |
| **Web of Science** | Topic (*Virtual Reality* OR *Virtual Reality Exposure Therapy* OR *Virtual Reality Immersion Therapy* OR *Exergaming* OR *Immersive Virtual Reality*) AND Topic (*Episiotomy* OR *Embryo Transfer* OR *Hysteroscopy* OR *Colpotomy* OR *Uterine Endoscopy* OR *Uteroscopy* OR *Colposcopy*OR *Culdoscopy*) |
| **CINAHL Complete** | AB(Virtual Reality OR Virtual Reality Exposure Therapy OR Exergaming OR immersive virtual reality OR non-immersive virtual reality) AND AB(Episiotomy OR Embryo Transfer OR Hysteroscopy OR Colpotomy) |
| **PEDro** | Virtual Reality AND Hysteroscopy  Virtual Reality AND Episiotomy  Virtual Reality AND Colposcopy  Virtual Reality AND Uteroscopy  Virtual Reality AND Colpotomy |

**Figure S1.** Overall biases identified with Cochrane Risk of Bias Tool

**Figure S2.** Funnel plot of the effectiveness of IVR in reducing after GOIP
